# Molecular study of HBZ and gp21 Human T cell lymphotropic virus type 1 proteins isolated from different clinical profile infected individuals

**DOI:** 10.1186/1742-4690-11-S1-P84

**Published:** 2014-01-07

**Authors:** Aline CA Mota-Miranda, Fernanda K Barreto, Everton Baptista, Joana P Monteiro-Cunha, Lourdes Farre-Valve, Bernardo Galvao-Castro, Luiz CJ Alcantara

**Affiliations:** 1 CPqGM/FIOCRUZ, Salvador, Bahia, Brazil; 2 EBMSP, Salvador, Bahia, Brazil; 3 UFBA, Salvador, Bahia, Brasil

## 

The HTLV-1 gp21 glycoprotein is involved in envelope trafficking and membrane targeting while bZIP protein is indispensable for cell growth and proliferation. This study aimed to assess the molecular diversity of gp21 and HBZ proteins, in TSP/HAM and healthy carriers. DNA samples from HTLV-1 infected individuals were submitted to PCR, sequencing, and the molecular analyses were performed using bioinformatics tools. From eight gp21 analyzed sequences one amino acid change (Y477H) was associated to the switch of helix to coil structure at secondary structure prediction. From ten HBZ analyzed sequences, two amino acid changes were identified (S9P and T95I) at the activation domain. One mutation (R112C) located at nuclear localization signal was present in 66.7% and 25% of HC and TSP/HAM groups, respectively. This is the first report of mutations in HBZ region. These polymorphisms might be important for viral fitness.

